# The cost of high cognitive insight: chain mediation of stigma and dysfunctional sleep beliefs and attitudes in chronotype among patients with schizophrenia

**DOI:** 10.3389/fpsyt.2026.1744418

**Published:** 2026-03-13

**Authors:** Shiyu You, Ying Xie, Xiaohan Wang, Shihan Tang, Dongmei Wu

**Affiliations:** 1 Chengdu University of Traditional Chinese Medicine, School of Nursing, Chengdu, China; 2 Department of Nursing, Sichuan Provincial People’s Hospital, School of Medicine, University of Electronic Science and Technology of China, Chengdu, China

**Keywords:** chronotype, cognitive insight, dysfunctional sleep beliefs and attitudes, mediation, mental health, schizophrenia, stigma

## Abstract

**Objective:**

This study examines the chain mediation effect through which cognitive insight impacts chronotype (eveningness chronotype tendency) via stigma and dysfunctional sleep beliefs and attitudes in patients with schizophrenia.

**Methods:**

Community-followed patients were sampled cross-sectionally (N = 785). Assessment tools included the Beck Cognitive Insight Scale, Attitudes Towards Mental Health Scale, Dysfunctional Beliefs and Attitudes about Sleep Scale, and Reduced Morningness-Eveningness Questionnaire, with demographic and treatment variables controlled. PROCESS Model 6 and 5000 bootstrapped estimates were used to assess indirect effects (standardized *β*, 95% CI), alongside collinearity and robustness diagnostics.

**Results:**

The correlation analysis indicated significant relationships among cognitive insight, chronotype, stigma, and dysfunctional sleep beliefs and attitudes (all *p* <.05). The chain mediation analysis revealed a significant total indirect effect (*β* = −0.083, 95% CI [−0.116, −0.054]). Three indirect pathways were confirmed: Stigma mediated the relationship between cognitive insight and sleep chronotype (*β* = −0.064, 95% CI [−0.093, −0.039]). Dysfunctional sleep beliefs and attitudes served as a mediator in the relationship between cognitive insight and sleep chronotype (*β* = −0.012, 95% CI [−0.028, −0.001]). Both stigma and dysfunctional sleep beliefs and attitudes acted as a chain mediator between cognitive insight and sleep chronotype (*β* = −0.007, 95% CI [−0.013, −0.002]). The direct effect was weakened and marginally significant (β = −0.070, 95% CI [−0.136, 0.000]).

**Conclusion:**

Higher cognitive insight is potentially associated with increased stigma and enhanced dysfunctional sleep beliefs and attitudes, and these factors are collectively correlated with eveningness chronotype tendency. Given the genetic and correlational evidence that morningness chronotype is associated with better mental health and lower schizophrenia risk, future interventions could focus on enhancing cognitive insight while addressing stigma and reconstructing sleep cognition/rhythm-light management, which may lead to a tendency toward morningness chronotype.

## 1 Introduction

The sleep-wake cycle oscillates approximately every 24 hours (1). It is mainly categorized into “morningness chronotype,” characterized by early sleep and wake times, and “eveningness chronotype,” described by late sleep and wake times (2). Sleep-wake rhythm disturbances are prevalent in patients with schizophrenia, with approximately 80% experiencing varying degrees of circadian rhythm abnormalities (3). Such disturbances are common prodromal symptoms of schizophrenia and are closely related to the severity of the disease symptoms (4), cognitive deficits (5), and impaired social functioning (6). Chronotype is a phenotype of circadian rhythm (7), and a significant tendency toward eveningness chronotype is often observed in the schizophrenia population (8). This chronotype is associated with higher levels of depression, poorer mental health and social adjustment, and impaired cardiometabolic regulation (9–11). Conversely, morningness chronotype has protective effects against various mental disorders, including schizophrenia (12). A study with a sample size of 700,000 indicated a correlational relationship between morningness chronotype and greater subjective well-being and lower schizophrenia risk, which supports “promoting morningness chronotype” as an intervention target (2). Thus, improving chronotype (promoting a shift towards morningness chronotype) may be a potential intervention target for alleviating schizophrenia symptoms and enhancing recovery (13).

To explore the psychological mechanisms of chronotype, we focus on cognitive insight.Cognitive insight is an important indicator of recovery in schizophrenia (14). It refers to the ability of individuals to reflect on abnormal experiences and correct erroneous interpretations, encompassing two dimensions: self-reflectiveness and self-certainty (15). The insight paradox in schizophrenia reveals that illness awareness is not a purely clinically beneficial factor: high insight is associated with improved treatment adherence, yet often accompanied by low self-esteem, hopelessness and depressive symptoms in the presence of internalized stigma; low insight is linked to impaired social function, but rarely involves psychological distress caused by stigma (16, 17). Internalized stigma acts as a core moderator of this paradox, and patients with high insight combined with moderate to severe internalized stigma exhibit the most significant impairments in psychological well-being and social function (16, 18), providing a key theoretical framework for analyzing the dual effects of cognitive insight (16–18). This suggests that the impact of cognitive insight on chronotype is not uniformly positive and underscores the need to examine the psychosocial mechanisms and the clinical double-edged sword effect.

Stigma is a key mediating factor in the relationship between cognitive insight and chronotype in patients with schizophrenia. A systematic review revealed that stigma is prevalent among individuals with schizophrenia (19). Stronger cognitive insight in patients often leads to more pronounced stigma; concurrently, higher self-certainty in their judgments makes them more likely to internalize negative stereotypes about mental illness, further exacerbating stigma (20). Stigma may be associated with social withdrawal and reduced exposure to sunlight, which could weaken external “time cues” and potentially lead to a tendency toward eveningness chronotype (21, 22). This pathway aligns with the social zeitgeber theory, which posits that disruption of social rhythmic cues can trigger and maintain biological clock phase misalignment (23, 24). Based on existing evidence, this study preliminarily hypothesizes that stigma may mediate the relationship between cognitive insight and chronotype.

In addition to psychosocial mechanisms, factors at the cognitive-behavioral level may play a significant role in maintaining an eveningness chronotype tendency. Harvey’s cognitive model of insomnia (25)suggests that catastrophic beliefs about sleep, excessive worry, and safety behaviors can lead to an overactivation of the sleep-wake system, thereby perpetuating insomnia and rhythm disturbances. Dysfunctional beliefs and attitudes about sleep are crucial cognitive factors influencing sleep behaviors, and elevated levels are associated with more severe insomnia symptoms (25–27). In patients with schizophrenia, there is limited research directly examining their dysfunctional sleep beliefs and attitudes. However, some studies indicate that individuals with psychosis often have impaired cognitive insight, manifested as high self-certainty and difficulty in correcting distorted beliefs, coupled with insufficient self-reflection (28). Thus, it is reasonable to hypothesize that the state of cognitive insight likely affects the formation and reinforcement of dysfunctional sleep beliefs in this patient group, although this mechanism requires empirical validation. Compared to morning-type individuals, those with an evening chronotype exhibit more pronounced dysfunctional sleep beliefs and attitudes (29). Evening-type individuals also tend to be less satisfied with their sleep duration and perceive sleep pattern changes as more challenging (30). Additionally, an increase in dysfunctional sleep beliefs and attitudes correlates with the severity across all insomnia subtypes, and an eveningness chronotype tendency is significantly associated with difficulty initiating sleep (26). Based on the existing evidence, this study preliminarily hypothesizes that dysfunctional sleep beliefs and attitudes may mediate the relationship between cognitive insight and chronotype.

Currently, research specifically addressing stigma and dysfunctional sleep beliefs and attitudes is relatively scarce. However, existing studies have provided some foundation for their potential association and subsequent hypotheses. Systematic review indicate that self-stigma, perceived stigma, and anticipated stigma are significantly associated with insufficient sleep, difficulties in falling asleep, and poor subjective sleep quality (31). Specifically, self-stigma is the progressive internalization of social negative stereotypes of mental illness by individuals with psychiatric disabilities that results in self-esteem decrement (32). Perceived stigma is subjective awareness of being devalued and discriminated against by society due to a specific social label one bears (33). Anticipated stigma represents the expectation of future discrimination or rejection, often leading to social withdrawal (34). Research shows that stigma leads to negative cognitive activities such as low self-esteem and negative expectations for health, and negative cognitive activities can lead to catastrophic interpretations of sleep consequences which align closely with irrational beliefs found in the Dysfunctional Beliefs and Attitudes About Sleep Scale, such as the belief that one must sleep a specific number of hours to function properly (21, 25, 35). Clinically, patients with chronic insomnia report higher levels of stigma, which coexists with poorer sleep quality and mental health, suggesting that stigma may delay help-seeking by amplifying dysfunctional sleep beliefs and attitudes (36). Based on this, we hypothesize that stigma and dysfunctional sleep beliefs and attitudes may mediate the relationship between cognitive insight and sleep chronotype.

In summary, this study proposes a chain mediation model based on the social zeitgeber theory and the cognitive model of insomnia to examine the mediating roles of stigma and dysfunctional sleep beliefs and attitudes in the relationship between cognitive insight and chronotype. The study presents the following hypotheses: 1) In patients with schizophrenia, cognitive insight level is negatively correlated with chronotype; specifically, higher cognitive insight is associated with a tendency towards the evening chronotype. 2) Stigma mediates the relationship between cognitive insight and chronotype. 3) Dysfunctional sleep beliefs and attitudes mediate the relationship between cognitive insight and chronotype. 4) Stigma and dysfunctional sleep beliefs and attitudes provide a chain mediation effect in the relationship between cognitive insight and chronotype. Elucidating this chain mechanism will not only deepen the theoretical understanding of the “insight paradox” but also offer empirical support for future interventions that aim to enhance cognitive insight, combined with destigmatization and cognitive sleep interventions, to improve the chronotype in patients with schizophrenia.

## 2 Methods

### 2.1 Participants and study design

This study was approved by the Ethics Committee of Chengdu Fourth People’s Hospital ([2017] Ethical Review No. (16)) and was registered in the Chinese Clinical Trial Registry (ChiCTR1800015219). A cross-sectional design was used, focusing on patients with schizophrenia from the Pengzhou community in Chengdu.

A total of 900 questionnaires were distributed via convenience sampling, and 818 were returned, corresponding to a response rate of 90.89%. After excluding 33 invalid questionnaires, 785 valid questionnaires were retained, yielding an effective response rate of 95.97%.

All patients were diagnosed by at least two attending psychiatrists according to DSM-5 criteria. Inclusion criteria were: 1) meeting DSM-5 diagnostic criteria; 2) age between 18–60 years; 3) receiving community follow-up. Exclusion criteria included: 1) presence of other psychiatric or severe physical illnesses; 2) history of substance dependence. The use of antipsychotic drugs was not restricted in this study. Some patients received stable antipsychotic drug treatment, with 1 to 3 types of psychotropic drugs used as part of community treatment. Therefore, the sample included patients with different drug use statuses.

Questionnaires were distributed and collected through the Wenjuanxing platform, a widely used online survey tool in China for electronic design, distribution and data collection of questionnaires. Psychiatrists and community nurses identified potentially eligible patients from their registration lists according to the inclusion and exclusion criteria. Community staff contacted eligible patients and their families to explain the research purpose and procedures. For those who initially expressed interest, face-to-face meetings were arranged to obtain written informed consent. Community staff then assisted participants in filling out the questionnaires through the Wenjuanxing platform on their personal smartphones, or provided tablet computers to ensure that participants with limited digital literacy could also participate.

### 2.2 Measures

#### 2.2.1 Cognitive insight

The Beck Cognitive Insight Scale (BCIS) measures “self-reflectiveness/self-certainty” and a composite index (reflectiveness-certainty), which reflects the ability to reflect on and correct abnormal experiences (15). The scale consists of 15 items scored on a 4-point Likert scale (0-3) and includes two subscales: self-reflectiveness and self-certainty. The total score is calculated by subtracting the self-certainty score from the self-reflectiveness score; higher scores indicate better overall cognitive insight. Recently, it has been applied among first-episode/high-risk psychiatric populations (37). The Chinese version has good reliability (self-reflectiveness: *α* = 0.70; self-certainty: *α* = 0.72) and is widely used (38).

#### 2.2.2 Stigma

The Attitudes Towards Mental Health Scale (ATMHP) assesses stigma and shame associated with mental health issues. This scale consists of five dimensions with a total of 35 items: attitudes toward psychological problems (8 items), external shame/stigma awareness (10 items), internal shame (5 items), familial shame (7 items), and personal shame (5 items). Each item is rated on a 4-point Likert scale (0-3), and higher total scores indicate stronger feelings of shame. The scale has demonstrated strong psychometric properties in populations with mental disorders (*α* = 0.94) (39) and has shown good reliability when used among Asian individuals (*α* = 0.85) (40).

#### 2.2.3 Dysfunctional beliefs and attitudes about sleep

Dysfunctional Beliefs and Attitudes about Sleep Scale (DBAS-16) evaluates sleep-related misconceptions such as “sleep catastrophizing” and “lack of control” (41). This study used the authorized Chinese version of the DBAS-16 developed by Fu with permission from the original author. This tool consists of 16 items rated on a 5-point Likert scale (1-5), with total scores ranging from 16 to 80. It is important to note that the Chinese version of DBAS-16 employs reverse scoring compared to the original English version: lower scores indicate more severe dysfunctional beliefs and attitudes about sleep, while higher scores indicate more adaptive sleep beliefs. The Chinese version has demonstrated good reliability (*α* = 0.77) (42).

#### 2.2.4 Chronotype

The Reduced Morningness-Eveningness Questionnaire (rMEQ) assesses an individual’s circadian rhythm or chronotype (43). The scale is a shortened version of the Morningness-Eveningness Questionnaire (MEQ) and consists of 5 items, scored using a mixed system ranging from 1 to 6, with total scores from 4 to 25. Lower scores suggest a tendency towards eveningness, indicating a predisposition for delayed sleep phase. Conversely, higher scores indicate a tendency towards morningness. It has been validated in populations with psychiatric disorders (44), and the Chinese version has shown acceptable measurement properties in previous applications (*α* = 0.70) (45).

#### 2.2.5 Demographic and clinical characteristics

Data on gender, residence, employment status, perceived family economic status, smoking habits, marital status, antipsychotic medication use in the past three months, psychotherapy, and Modified Electroconvulsive Therapy (MECT) were collected.

### 2.3 Statistical analysis methods

Statistical analyses were conducted using SPSS 25.0. Continuous variables with normal or near-normal distributions were presented as mean ± standard deviation (Mean ± SD), while those with skewed distributions are described using median and quartiles. Categorical variables are expressed as frequencies and percentages. Normality of continuous variables was assessed with the Shapiro-Wilk test. Group comparisons were performed using analysis of variance (ANOVA) or the Kruskal-Wallis test. Variables significantly associated with chronotype (*p* <.05) were selected as control variables, and multicollinearity was checked with a Variance Inflation Factor (VIF < 5) before inclusion in the model. Relationships among variables were analyzed using Spearman correlation, and stepwise regression was employed to identify significant influencing factors. To verify the serial mediation effect of stigma and dysfunctional sleep beliefs and attitudes between cognitive insight and chronotype, mediation analysis was performed using Model 6 in the SPSS PROCESS macro. The bootstrap sampling method, with 5,000 resamples, was used to compute 95% confidence intervals. A mediation effect was considered statistically significant if the 95% confidence interval did not contain zero.

## 3 Results

### 3.1 Common method bias test

Before conducting formal data analysis, Harman’s single-factor test was performed via exploratory factor analysis (EFA). Results revealed that the variance explained by the initial factor—with an eigenvalue exceeding one—stood at 39.852%, falling below the 40.000% threshold. This finding indicates that no significant common method bias is present in the data.

### 3.2 Univariate analysis of demographic and clinical characteristics and chronotype in community schizophrenia patients

**Table 1** summarizes the general characteristics of 785 community-dwelling patients with schizophrenia, among whom 414 (52.7%) are male and 371 (47.3%) are female. Over the past year, 85.2% of the patients resided in rural areas, 70.7% were currently unemployed, 56.1% rated their family’s economic status as average, 72.5% had never smoked, and 47.6% were married. Additionally, 83.2% had used antipsychotic medication in the preceding three months, 96.3% had not undergone psychotherapy, and 99.1% had not received modified electroconvulsive therapy (MECT). **Table 1** also illustrates variations in chronotype across demographic and clinical characteristics. Significant correlations were observed between chronotype and past-year residence, employment status, marital status, receipt of psychotherapy, and MECT exposure. These covariates will therefore be incorporated as control variables in subsequent models. Use of antipsychotic medication in the past three months was not significantly associated with chronotype and was therefore not included as a covariate.

**Table 1:** Demographic, clinical characteristics, and univariate analysis of participants (N = 785).

Variables	*n* (%)	Chronotype	
		*t* or *F*	*p*
Gender		(783) = -0.58	0.564
male	414 (52.7)		
female	371 (47.3)		
Residence		(2, 782) = 7.31	0.001
rural	669 (85.2)		
town	86 (11.0)		
city	30 (3.8)		
Employment Status		(3, 781) = 2.77	0.041
studying	2 (0.3)		
employed	91 (11.6)		
unemployed	555 (70.7)		
non-labor force	137 (17.5)		
Perceived Household Economic Status		(4, 780) = 2.08	0.081
very poor	85 (10.8)		
relatively poor	231 (29.4)		
average	440 (56.1)		
relatively good	28 (3.6)		
very good	1 (0.1)		
Smoking Status		(2, 782) = 0.42	0.659
never smoked	569 (72.5)		
currently smoking	205 (26.1)		
quitting/former Smoker	11 (1.4)		
Marital Status		(4, 780) = 3.85	0.004
single	258 (32.9)		
married	374 (47.6)		
divorced	127 (16.2)		
widowed	18 (2.3)		
other	8 (1.0)		
Use of Antipsychotic Medication in the Past 3 Months		(783) =-0.83	0.409
yes	653 (83.2)		
no	132 (16.8)		
Psychological Therapy in the Past 3 Months		(783) =3.22	0.001
yes	29 (3.7)		
no	756 (96.3)		
MECT Status in the Past 3 Months		(783) = -2.09	0.037
yes	7 (0.9)		
no	778 (99.1)		

### 3.3 Correlation analysis of cognitive insight, stigma, dysfunctional sleep beliefs and attitudes, and chronotype

As presented in **Table 2**, cognitive insight was positively correlated with stigma (*r* = 0.251, *p* = .000) and negatively correlated with DBAS-16 scores (*r* = -0.090, *p* = .012), as well as with rMEQ scores (*r* = -0.139, *p* = .000). Given that lower DBAS-16 scores indicate more severe dysfunctional sleep beliefs, and lower rMEQ scores indicate a stronger eveningness tendency, these negative correlations suggested that higher cognitive insight was associated with more severe dysfunctional sleep beliefs and a stronger eveningness tendency. Stigma exhibited negative correlations with both DBAS-16 scores (*r* = -0.106, *p* = .003) and rMEQ scores (*r* = -0.269, *p* = .000). These negative correlations indicated that higher stigma was associated with more severe dysfunctional sleep beliefs (lower DBAS-16 scores) and a stronger eveningness tendency (lower rMEQ scores). Meanwhile, DBAS-16 scores were positively correlated with rMEQ scores (*r* = 0.249, *p* = .000). Considering the scoring of both scales, this positive correlation indicated that more severe dysfunctional sleep beliefs (lower DBAS-16 scores) were associated with a stronger eveningness tendency (lower rMEQ scores).

**Table 2:** Correlation of cognitive insight, stigma, dysfunctional sleep beliefs and attitudes, and chronotype.

	M ± SD	1	2	3	4
cognitive insight	3.30 ± 1.59	1.00			
stigma	25.64 ± 24.07	0.251***	1.00		
DBAS	42.54 ± 10.89	-0.090*	-0.106**	1.00	
chronotype	17.36 ± 2.45	-0.139***	-0.269***	0.249***	1.00

*p <.05, **p <.01, ***p <.001.

### 3.4 Analysis of chain mediation effects

Prior to conducting mediation analysis, multicollinearity diagnostics were performed for all independent and control variables. Variance inflation factor (VIF) values ranged from 1.008 to 1.374, all below the critical threshold of 5. This finding suggested the absence of multicollinearity among these variables. Consequently, it validated that these data were appropriate for subsequent regression analysis and mediation effect analysis. Regression analysis results demonstrated that cognitive insight exerted a direct positive predictive effect on stigma (*β* = 0.301, *p* <.001). It also had a direct negative predictive effect on DBAS-16 scores (*β* = -0.098, *p* <.01) and rMEQ scores (*β* = -0.153, *p* <.001). Given the scoring of these scales, these negative coefficients indicate that higher cognitive insight is associated with more severe dysfunctional sleep beliefs (lower DBAS-16 scores) and a stronger eveningness tendency (lower rMEQ scores). Additionally, stigma was found to negatively predict DBAS-16 scores (*β* = -0.172, *p* <.001) and rMEQ scores (*β* = -0.213, *p* <.001). These negative coefficients indicate that higher stigma is associated with more severe dysfunctional sleep beliefs (lower DBAS-16 scores) and a stronger eveningness tendency (lower rMEQ scores). In contrast, DBAS-16 scores exhibited a direct positive predictive effect on rMEQ scores (*β* = 0.127, *p* <.001). Considering the scoring of both scales, this positive coefficient indicates that more severe dysfunctional sleep beliefs (lower DBAS-16 scores) are associated with a stronger eveningness tendency (lower rMEQ scores). Detailed regression coefficients are presented in **Table 3**.

**Table 3:** Chain mediation model of cognitive insight, stigma, dysfunctional sleep beliefs and attitudes, and chronotype: a regression analysis.

Regression equation		Fitting index			Regression coefficient				
Result variable	predictor variable	*R*	*R^2^*	*F*	*β*	SE	*t*	LLCI	ULCI
stigma	cognitive insight	0.390	0.152	23.236***	0.301	0.317	9.072***	2.251	3.493
DBAS	cognitive insight	0.290	0.084	10.179***	-0.098	0.157	-2.688**	-0.729	-0.114
	stigma				-0.172	0.017	-4.626***	-0.111	-0.045
chronotype	cognitive insight	0.363	0.132	14.719***	-0.070	0.035	-1.974*	-0.136	0.000
	stigma				-0.213	0.004	-5.772***	-0.029	-0.014
	DBAS				0.127	0.008	3.637***	0.013	0.044
chronotype	cognitive insight	0.265	0.070	9.810***	-0.153	0.034	-4.406***	-0.215	-0.082

1.*p <.05, **p <.01, ***p <.001.

2. All models controlled for covariates such as place of residence, employment status, marital status, psychological therapy, and MECT.

**Figure 1:**
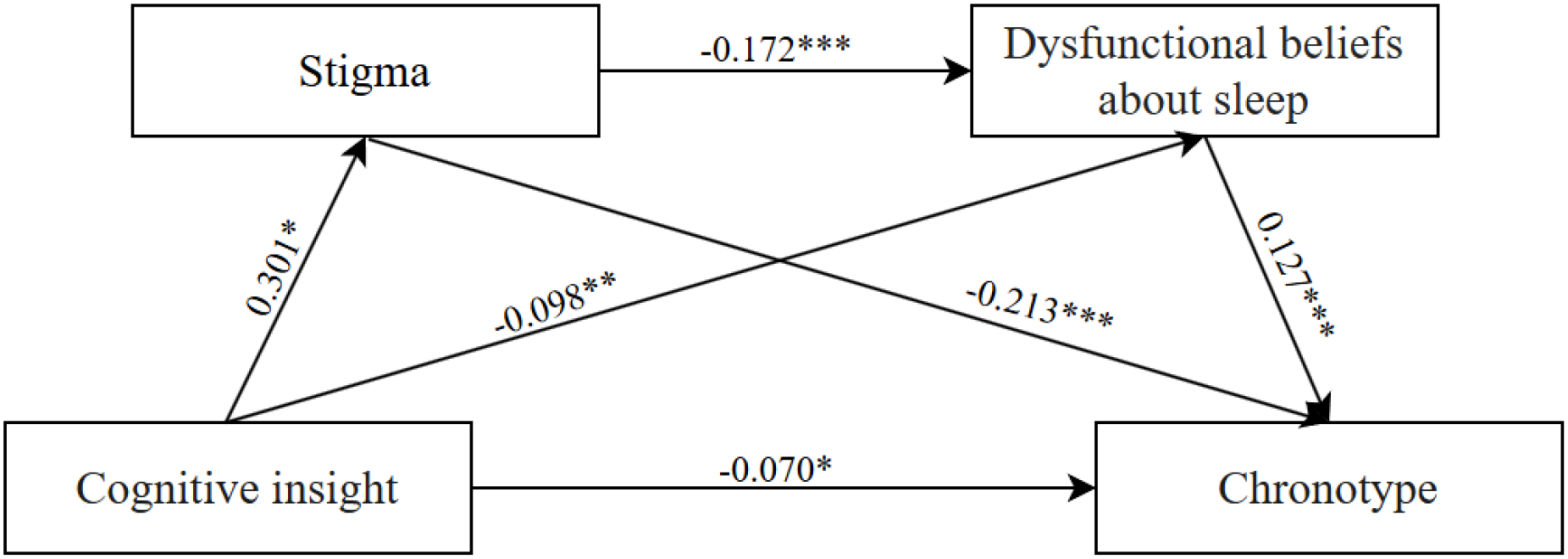
Mediation model of cognitive insight on chronotype.

As presented in **Table 4** and **Figure 1**, stigma and dysfunctional sleep beliefs and attitudes function as sequential mediators in the relationship between cognitive insight and chronotype. Both direct and total effects were significant, supporting a partial mediation model. All three mediation pathways reached statistical significance, confirming the existence of a chain mediation effect: cognitive insight not only exerts a direct influence on chronotype but also impacts it indirectly through individual and sequential mediation via stigma and dysfunctional sleep beliefs and attitudes. Specifically, among the three mediation pathways: The mediating effect of stigma in the cognitive insight–chronotype relationship was -0.064 (95% CI [-0.093, -0.039]), accounting for 41.83% of the total effect; The mediating effect of dysfunctional sleep beliefs and attitudes in this relationship was -0.012 (95% CI [-0.028, -0.001]), representing 7.84% of the total effect; This negative indirect effect indicates that higher cognitive insight is associated with more severe dysfunctional sleep beliefs (lower DBAS-16 scores), which in turn is associated with a stronger eveningness tendency (lower rMEQ scores). The chain mediating effect of stigma and dysfunctional sleep beliefs and attitudes (in sequence) was -0.007 (95% CI [-0.013, -0.002]), contributing 4.58% of the total effect. This negative chain effect indicates that higher cognitive insight is associated with increased stigma, which is associated with more severe dysfunctional sleep beliefs, ultimately associated with a stronger eveningness tendency. Notably, the indirect effect via the stigma-only pathway (-0.064) was larger than that of the chain mediation pathway (-0.007) and the dysfunctional sleep beliefs and attitudes-only pathway (-0.012). The total indirect effect was -0.083 (95% CI [-0.116, -0.054]), accounting for 54.25% of the total effect, indicating that cognitive insight primarily influences chronotype through mediating pathways. Bootstrap 95% confidence intervals for all indirect effects did not include zero, verifying the statistical significance of these mediation effects.

**Table 4:** Examination of the chain mediation effect among cognitive insight, stigma, dysfunctional sleep beliefs and attitudes, and chronotype.

Path	Effect	Boot SE	95% CI	Relative mediation effect
total effect	-0.153	0.034	[-0.215,-0.082]	100%
direct effect	-0.070	0.035	[-0.136,0.000]	45.75%
total indirect effect	-0.083	0.016	[-0.116,-0.054]	54.25%
cognitive insight → stigma → DBAS → chronotype	-0.007	0.003	[-0.013,-0.002]	4.58%
cognitive insight → stigma → chronotype	-0.064	0.014	[-0.093,-0.039]	41.83%
cognitive insight →DBAS → chronotype	-0.012	0.007	[-0.028,-0.001]	7.84%

## 4 Discussion

### 4.1 Analysis of the chain mediation mechanism between cognitive insight and chronotype

This study first found that higher cognitive insight in patients with schizophrenia was associated with a stronger tendency toward eveningness chronotype, which is consistent with hypothesis H1. This finding aligns with the “insight paradox” theory, which suggests that an increase in cognitive insight does not always translate to improved functioning; instead, it may exacerbate specific health risks through negative psychological pathways (18). Mechanistically, higher cognitive insight allows patients to perceive the nature of their illness and their functional limitations more clearly. In a societal environment where mental illness is highly stigmatized, such awareness can easily be internalized as stigma and self-deprecation (21, 46, 47). Integrating the social zeitgeber theory, stigma-induced social withdrawal may reduce patients’ daytime social interactions and exposure to natural morning light, while potentially increasing nighttime use of electronic screens (exposure to blue light). According to the human phase response curve to light, this pattern of light exposure may be associated with a delay in circadian phase, which is ultimately linked to eveningness chronotype tendency (48).

The model analysis further revealed that stigma serves as the primary mediator in the relationship between cognitive insight and chronotype, thus consistent with hypothesis H2. Consistent with existing indirect evidence, cognitive insight was significantly related to stigma in samples of schizophrenia patients (49). Experiencing discrimination and self-stigmatization is associated with sleep disturbances in individuals with mental disorders (50). In our study sample, 85.2% of schizophrenia patients resided in rural areas, and 70.7% were unemployed, likely facing heightened social exclusion and self-stigma pressures. Research conducted in rural China indicated that the level of internalized stigma among individuals with severe mental illness was both widespread and severe (51). The relatively closed social environment in rural areas may amplify the negative effects of “diagnostic labeling” (52). Additionally, internalized stigma is significantly related to employment status (53). Under these dual pressures, the negative correlation between stigma and chronotype may be more pronounced. Cross-cultural evidence shows significant associations between stigma and depression, low self-esteem, poor quality of life, unfavorable attitudes toward medication, with non-urban/unemployed populations being at higher risk (54), consistent with the structure of our sample. Furthermore, even after controlling for covariates such as residence and employment status, the mediating effect of stigma remains significant. This suggests that the mediation effect of stigma is not confounded by demographic or clinical characteristics, further confirming its robustness as an independent psychological mechanism.

The study demonstrates that dysfunctional sleep beliefs and attitudes mediate the relationship between cognitive insight and chronotype, thereby consistently supporting hypothesis H3. Although the direct mediation effect of dysfunctional sleep beliefs and attitudes is small, the chain pathway remains significant. In clinical samples, higher cognitive insight helps individuals recognize and correct maladaptive beliefs such as “loss of control over sleep,” whereas lower cognitive insight may allow these beliefs to persist (55). These dysfunctional beliefs, through behaviors like “napping during the day” and “excessive anxiety before sleep,” are associated with disruptions in the sleep-wake cycle, which may correspond to deviations in chronotype, such as delayed chronotype (56). Within the schizophrenia population, cognitive behavioral therapy for insomnia (CBT-I) has shown that changing erroneous sleep-related beliefs and insomnia behaviors can improve sleep and provide additional benefits for clinical outcomes, such as positive symptoms. This further supports the notion of “sleep beliefs” as an actionable mediator (57).

Ultimately, the study suggests that enhanced insight is not an unequivocal benefit. When it coexists with stigma, it may be associated with emotional responses such as shame or self-deprecation, which in turn are correlated with disrupted sleep cognition via inducing catastrophizing and a lack of perceived control. This may contribute to behaviors like staying up late, lingering in bed, taking daytime naps, and using screens at night, which are associated with the phase delay characteristic of the evening chronotype. This is consistent with hypothesis H4 and aligns with the core view of Harvey’s cognitive model of insomnia, which posits that catastrophic sleep beliefs and maladaptive behaviors jointly maintain rhythmic disturbances (58, 59). In terms of the pathway mechanism, high cognitive insight is associated with increased stigma and may further be linked to the formation of dysfunctional sleep beliefs and attitudes: the anxiety induced by stigma may be related to an increased perceived threat of sleep failure, which may be accompanied by catastrophic cognitions such as “a single bad night of sleep will have severe consequences. (31, 60) These maladaptive beliefs may contribute to patients adopting maladaptive strategies such as staying up late to ensure complete fatigue and lingering in bed during the day to compensate (61). These behaviors are associated with disruptions in the stability of the sleep-wake rhythm (62) and may correspond to an eveningness chronotype tendency (63). It is noteworthy that only 3.7% of patients in this study sample have received psychological treatment, which echoes previous research indicating that, in the absence of professional interventions, dysfunctional sleep beliefs and attitudes are difficult to modify (64). The discovery of the chain mediation effect further indicates that dysfunctional sleep beliefs and attitudes do not act independently. Instead, they work together with stigma to form a transmission chain of emotion-triggered cognition. This finding provides theoretical support for integrated clinical interventions, including destigmatization, sleep cognitive correction, and rhythm-light management.

### 4.2 Clinical implications

In clinical practice, it is essential to include stigma and the dysfunctional sleep beliefs and attitudes as key mediating targets in assessment systems. Regular monitoring of these factors should be undertaken to evaluate their impact on chronotype interventions. Additionally, tailored interventions should be implemented based on individual characteristics, such as medication usage, light exposure environment, and initial chronotype. Combining psychological interventions with rhythm and light management can also be beneficial. In rural populations, an integrated approach delivered through primary health institutions could include anti-stigma campaigns, daytime activity promotion, and encouraging outdoor morning activities. For individuals with high scores on dysfunctional sleep beliefs and attitudes, integrating core modules of CBT-I, such as cognitive restructuring, stimulus control, and sleep restriction, can be used for intervention, with a focus on strengthening cognitive reappraisal components (65).

## 5 Strengths and limitations

On the strengths side, firstly, the large sample size derived from community follow-ups rather than inpatient acute phases reflects the association between cognitive insight and chronotype in patients under “everyday stable conditions,” offering more practical implications for community mental health services. Secondly, the study employed a chain mediation model with Bootstrap resampling to estimate indirect effects, clearly presenting the relative weight of each mediating pathway. Thirdly, multicollinearity diagnostics were conducted, and several sociodemographic and treatment variables were controlled, providing initial assurance of model stability.

Our study has several limitations: the overall effect size is small, and the cross-sectional design implies that the chain pathways identified should be interpreted as statistical associations rather than causal relationships. Self-reported measures may contain biases, and the reduced Morningness-Eveningness Questionnaire is subjective rather than objective. Additionally, the sample is from a single region, which limits generalizability. Additionally, we did not systematically collect comprehensive indicators for metabolic syndrome (e.g., waist circumference, fasting blood glucose, blood lipid levels, etc.), leading to an incomplete assessment of metabolic status. Key clinical information, including duration of illness, duration of untreated psychosis, and treatment adherence, was not collected, which may have potential impacts on the present findings.

## 6 Conclusion

In conclusion, this study adopts a cognitive perspective to construct and verify a chain mediation model. Beginning with cognitive insight, it sequentially incorporates stigma and dysfunctional sleep beliefs and attitudes, ultimately influencing sleep chronotype, revealing potential pathways linking cognitive insight and eveningness chronotype tendency in individuals with schizophrenia. Despite the limitations of the cross-sectional design and measurement tools, this study provides preliminary theoretical support for integrating cognitive interventions with rhythm adjustments into sleep or rhythm management for mental disorders. Future research could employ a longitudinal-intervention hybrid design, integrate objective rhythm measures, and utilize instrumental variable/Mendelian randomization or cross-lagged panel models to verify the temporal causal sequence of “stigma-sleep cognition-chronotype.” Future studies should also collect data on duration of illness, duration of untreated psychosis, and treatment adherence to better control for potential confounding effects. This approach has the potential to advance the scientific and precise intervention of circadian rhythms and sleep health in patients with schizophrenia.

## Data Availability

The original contributions presented in the study are included in the article. Further inquiries can be directed to the corresponding author.
